# Inequality in Mortality and Cardiovascular Risk Among Young, Low-Income, Self-Employed Workers: Nationwide Retrospective Cohort Study

**DOI:** 10.2196/48047

**Published:** 2024-09-20

**Authors:** Byungyoon Yun, Heejoo Park, Jaesung Choi, Juyeon Oh, Juho Sim, Yangwook Kim, Jongmin Lee, Jin-Ha Yoon

**Affiliations:** 1 Department of Preventive Medicine, Yonsei University College of Medicine, Seoul, Republic of Korea; 2 The Institute for Occupational Health, Yonsei University College of Medicine, Seoul, Republic of Korea; 3 Institute for Innovation in Digital Healthcare, Yonsei University Health System, Seoul, Republic of Korea; 4 Department of Public Health, Graduate School, Yonsei University, Seoul, Republic of Korea; 5 Department of Global Economics, Sungkyunkwan University, Seoul, Republic of Korea

**Keywords:** self-employed, employee, all-cause mortality, cardiovascular disease, mental illness, socioeconomic status, nationwide study, inequality, effect modification, health checkups

## Abstract

**Background:**

Self-employment is a significant component of South Korea’s labor force; yet, it remains relatively understudied in the context of occupational safety and health. Owing to different guidelines for health checkup participation among economically active individuals, disparities in health maintenance may occur across varying employment statuses.

**Objective:**

This study aims to address such disparities by comparing the risk of all-cause mortality and comorbidities between the self-employed and employee populations in South Korea, using nationwide data. We sought to provide insights relevant to other countries with similar cultural, social, and economic contexts.

**Methods:**

This nationwide retrospective study used data from the Korean National Health Insurance Service database. Participants (aged 20‐59 y) who maintained the same insurance type (self-employed or employee insurance) for ≥3 years (at least 2008‐2010) were recruited for this study and monitored until death or December 2021—whichever occurred first. The primary outcome was all-cause mortality. The secondary outcomes were ischemic heart disease, ischemic stroke, cancer, and hospitalization with a mental illness. Age-standardized cumulative incidence rates were estimated through an indirect method involving 5-unit age standardization. A multivariable Cox proportional hazards model was used to estimate the adjusted hazard ratio (HR) and 95% CI for each sex stratum. Subgroup analyses and an analysis of the effect modification of health checkup participation were also performed.

**Results:**

A total of 11,652,716 participants were analyzed (follow-up: median 10.92, IQR 10.92-10.92 y; age: median 42, IQR 35-50 y; male: n=7,975,116, 68.44%); all-cause mortality occurred in 1.27% (99,542/7,851,282) of employees and 3.29% (124,963/3,801,434) of self-employed individuals (*P*<.001). The 10-year cumulative incidence rates of all-cause mortality differed significantly by employment status (1.1% for employees and 2.8% for self-employed individuals; *P*<.001). The risk of all-cause mortality was significantly higher among the self-employed individuals when compared with that among employees, especially among female individuals, according to the final model (male: adjusted HR 1.44, 95% CI 1.42‐1.45; female: adjusted HR 1.89, 95% CI 1.84‐1.94; *P*<.001). The risk of the secondary outcomes, except all types of malignancies, was significantly higher among the self-employed individuals (all *P* values were <.001). According to subgroup analyses, this association was prominent in younger individuals with lower incomes who formed a part of the nonparticipation groups. Furthermore, health checkup participation acted as an effect modifier for the association between employment status and all-cause mortality in both sexes (male: relative excess risk due to interaction [RERI] 0.76, 95% CI 0.74‐0.79; female: RERI 1.13, 95% CI 1.05‐1.21).

**Conclusions:**

This study revealed that self-employed individuals face higher risks of all-cause mortality, cardio-cerebrovascular diseases, and mental illnesses when compared to employees. The mortality risk is particularly elevated in younger, lower-income individuals who do not engage in health checkups, with health checkup nonparticipation acting as an effect modifier for this association.

## Introduction

Self-employed individuals constitute a significant portion of the Republic of Korea’s economically active population, although this trend is declining. According to the OECD (Organisation for Economic Co-operation and Development), Korea’s self-employment rate was 23.91% in 2021 (rank 7) [1]. However, self-employed individuals remain overlooked in global occupational safety and health analyses [2]. Owing to self-employed individuals’ higher flexibility and precarity, previous studies predominantly focused on their mental health outcomes or self-rated health, obtaining inconsistent results. Some studies have reported increased job satisfaction and less emotional stress among self-employed individuals in comparison to those among employed workers [3,4]. However, other studies have found that self-employment is associated with emotional exhaustion, self-reported depression, anxiety, insomnia, suicidal behavior, and chronic conditions [5-9].

Comparing all-cause mortality is a simple way to examine health disparities between the self-employed and employee populations [10]. Previous studies examined this difference, but the results were inconsistent. Toivanen et al [11] conducted a study in Sweden, comparing mortality differences between self-employed individuals and employees, and reported a lower mortality risk among the self-employed individuals, with variations between the two groups depending on industry, sex, and legal form of self-employment. Willeke et al [12] conducted a meta-analysis of the Northern European population and found that self-employed individuals’ cardiovascular-related mortality risk was lower when compared to that of white-collar workers and higher when compared to that of blue-collar workers. With regard to Asian countries, some studies compared mortality risk based on employment statuses in the male and female Japanese populations. The results showed a significantly higher mortality risk among self-employed female individuals, but the difference was not significant among self-employed male individuals [13,14].

These inconsistent results might be due to cultural and social differences across varying countries. In Korea, a significant difference exists between the self-employed and employee populations with respect to the existence of responsible managers. The Korean government requires employers to implement periodic health checkups to maintain employees’ health statuses and prevent delayed diagnoses of fatal diseases, as per the *Occupational Safety and Health Act* [15]. However, there is no such obligation for the self-employed population. Consequently, disparities in health maintenance may occur owing to Korea’s legal characteristics, and investigating whether inequalities exist in health outcomes is crucial. Nevertheless, few studies have examined mortality risk and chronic comorbidities among the self-employed population in Korea. Lim et al [16] conducted a study on employment status and all-cause mortality among male Korean workers and reported that the self-employed workers (petty bourgeoisie) had a higher risk of mortality than employees; however, the study was limited in its representativeness and lacked detailed subgroup analyses, as a result of the small sample size.

This study aims to compare the mortality risk and comorbidities between the self-employed and employee populations in Korea, by using nationwide data, while considering social and occupational characteristics. To generalize our findings, we reviewed and synthesized comprehensive knowledge, explored cultural differences, and identified possible mechanisms that may contribute to the observed disparities. By examining these factors, we hope to provide valuable insights that are relevant to not only Korea but also other countries with similar cultural, social, and economic contexts.

## Methods

### Ethical Considerations

This study adhered to the ethical principles of the Declaration of Helsinki and was approved by the institutional review board of Yonsei University Hospital (approval number: IRB 4-2022-0813). As this was a retrospective cohort study of deidentified administrative data, the need for informed consent was waived.

### Data Set

The database of the National Health Insurance Service (NHIS) encompasses comprehensive data on the entire population of Korea—roughly 50 million individuals—spanning from 2002 to 2021. These include demographic and socioeconomic details, such as age, gender, employment, and income levels, as well as extensive medical records, including hospital and outpatient visits, procedures, and prescriptions categorized by the *International Statistical Classification of Diseases, Tenth Revision* (*ICD-10*) codes. Additionally, the database contains records of individuals who participated in health checkups, including outcomes of general health assessments and lifestyle behavior surveys [17]. In the Republic of Korea, article 19 of the *Act on Prohibition of Age Discrimination in Employment and Elderly Employment Promotion* stipulates that employers should set employees’ retirement age at 60 years or older [18]. Korea’s NHIS also categorizes citizens based on insurance type—self-employed householders and their dependents, employee householders and their dependents, and medical aid recipients and their dependents—according to chapter II of the *National Health Insurance Act* [19]. This retrospective cohort study enlisted working individuals who were either self-employed householders or employee householders, were aged 20 to 59 years, and maintained the same insurance type (self-employed or paid employee insurance) for ≥3 years (at least 2008‐2010).

The index date was defined as January 1, 2011, and a washout period (2002‐2010) was used to exclude individuals with fatal diseases. The exclusion criteria were defined as follows: (1) missing data values, including those for region and income; (2) history of malignancy; (3) history of ischemic heart disease; (4) history of ischemic stroke; and (5) occurrence of all-cause mortality within 1 year of the index date.

### Outcomes and Follow-Up

All-cause mortality was the primary end point of this study. The incidence of any type of malignancy (excluding thyroid cancer), ischemic heart disease, ischemic stroke, and hospitalization due to a mental illness was defined as a secondary outcome. All diseases were defined by using the *ICD-10* codes (all malignancy types: all *C* codes except C73; hospitalization due to mental illness: F00-F69; ischemic heart disease: I21-I23 and I25; ischemic stroke: I63-I64). The diagnosis date of each disease was defined as the date of the earliest hospitalization or outpatient visit for individuals with at least 1 hospitalization or 3 outpatient visits related to the disease. Participants were monitored until death or December 31, 2021—whichever occurred first. For the analyses of secondary outcomes, participants were evaluated based on the incidence of target diseases.

### Other Variables

**Figure 1.**
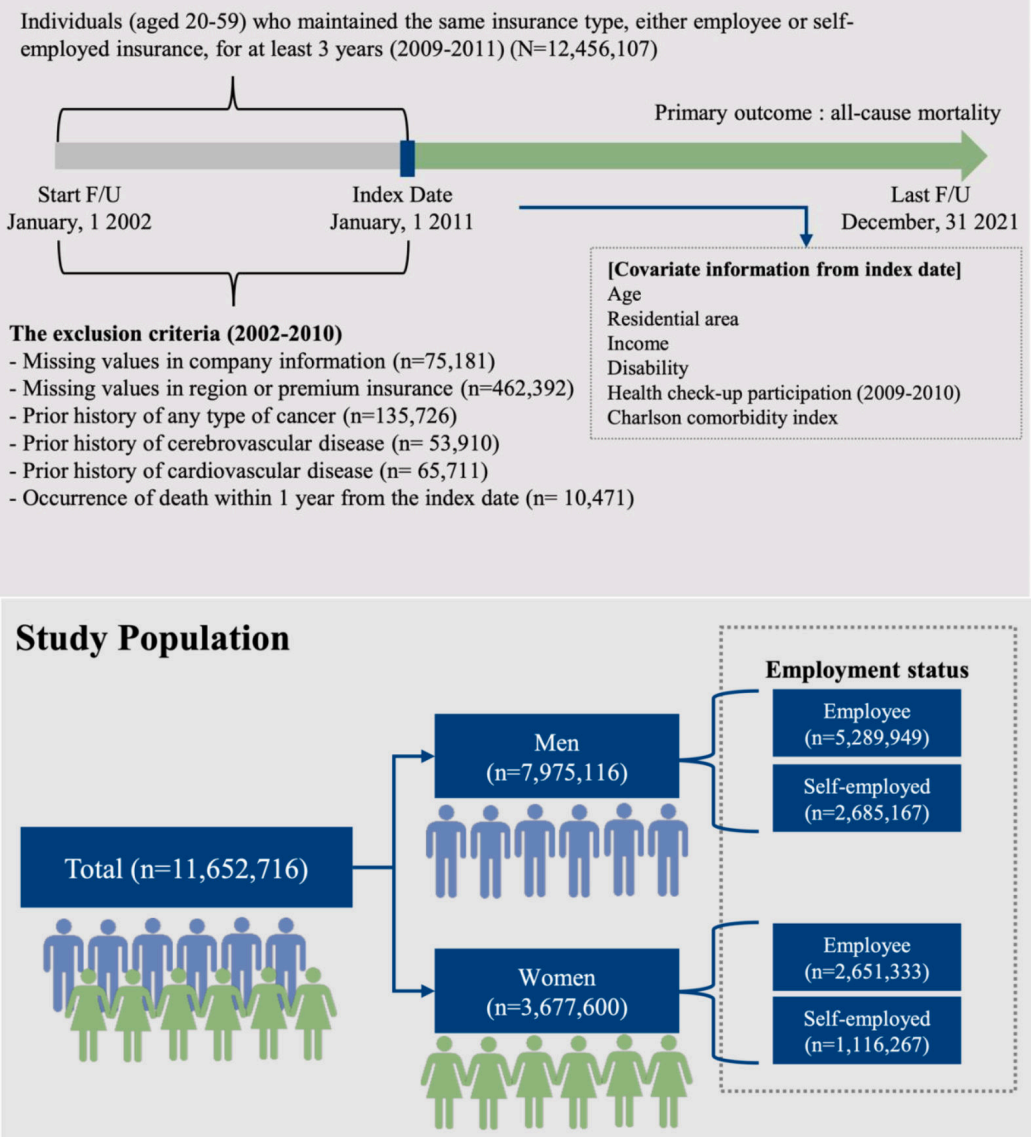
Schematic flow of the selection and follow-up processes for working individuals aged 20 to 59 y in the retrospective cohort, who were recruited from 2008 to 2010 (sourced from the National Health Insurance Service database in Korea; n=11,652,716). A washout period for diseases was included in the Charlson Comorbidity Index (2002-2010). F/U: follow-up.

Participants were stratified into the self-employed and employee groups based on their employment status, which was determined based on their insurance type for the 3 years preceding their inclusion in the study. As per government recommendation, the National Health Check-up Service should be completed every 2 years by all individuals, during which each participant’s physical attributes, including BMI, waist circumference, blood pressure, blood and urine test results, and chest x-ray results, are measured, and several health-related questionnaires are filled out. Nonparticipation and participation groups referred to those who did not undergo a health checkup between 2009 and 2010 and those who did undergo at least 1 health checkup during this period, respectively. All individuals were divided into quartiles based on their income level, which was indirectly determined by their NHIS insurance premium. According to the Korean government’s disability registration system, which was implemented in 1988, individuals’ disability severity is classified into 1 of 6 levels depending on the degree of functional loss [20]. In this study, participants were divided into the following two categories: those without disabilities and those with a disability of any grade. Participants’ residential areas were categorized as either “metropolitan” or “other.” Furthermore, participants were divided into 3 groups based on their Charlson Comorbidity Index (CCI) scores (0, 1, and ≥2) from the index year. The CCI score was calculated based on a minimum of 1 hospitalization or 3 outpatient visits that were associated with each category of the CCI and occurred within the period of 2002 to 2010. The categories used for calculating the CCI score are summarized in Multimedia Appendix 1. The schematic flow of the study design is described in Figure 1.

### Statistical Analysis

The baseline characteristics of all participants—stratified by insurance type and sex—were expressed by frequencies with percentages or medians with IQRs. The age-standardized cumulative incidence rates of all-cause mortality and secondary outcomes were estimated by using an indirect method involving 5-unit age standardization. The reference population was derived from the 2010 census population in Korea. Adjusted hazard ratios (HRs) and 95% CIs of the outcomes, by insurance type, were estimated by using multivariable Cox proportional hazard models. Model 1 was adjusted for age; model 2 was adjusted for socioeconomic status, including residential area, income, and disability, in addition to age; and model 3 was adjusted for health checkup participation and CCI score, in addition to the factors for which models 1 and 2 were adjusted. Furthermore, subgroup analyses were conducted by age group, region, income level, disability, and CCI score. All analyses were stratified by sex.

The risk of secondary outcomes for the self-employed individuals and that for paid employees were compared, stratified by sex, and estimated by using a multivariable Cox regression model. For the sensitivity analysis, the risk of all-cause mortality, by insurance type, was estimated with additional time lags of 2, 3, and 4 years. Additionally, the effect modification of health checkup participation on the association between employment status and all-cause mortality was examined with the relative excess risk due to interaction (RERI). A *P* value of <.05 was regarded as statistically significant. All statistical analyses were performed by using R version 4.0.3 (R Foundation for Statistical Computing) and SAS Enterprise version 8.2 (SAS Institute).

## Results

### Participants’ Baseline Characteristics According to Employment Status

Of the 12,456,107 participants, 11,652,716 remained after exclusion (Figure 1). The median age of participants was 42 (IQR 35-50) years, and 68.44% (7,975,116/11,652,716) were male. According to the baseline characteristics of participants, who were stratified by employment status and sex, the self-employed group had significantly higher proportions of older individuals; nonparticipation in health checkups; and participants with low incomes, disability, and high CCI scores, regardless of sex (all *P* values were <.001; Table 1). Female participants tended to be younger, participated more in health checkups, earned lower incomes, and had lower disability levels when compared to male participants.

**Table 1.** Baseline characteristics of working individuals aged 20 to 59 y in the retrospective cohort, who were recruited from 2008 to 2010 (sourced from the National Health Insurance Service database in Korea; n=11,652,716).

Characteristics		Male (n=7,975,116)		Female (n=3,677,600)	
		Employee (n=5,289,949)	Self-employed (n=2,685,167)	Employee (n=2,561,333)	Self-employed (n=1,116,267)
Age (y), median (IQR)		41 (35‐48)	47 (41‐52)	38 (31‐46)	46 (39‐52)
**Age group (y), n (%)**					
	20s	351,229 (6.64)	69,382 (2.58)	513,801 (20.06)	56,714 (5.08)
	30s	1,926,971 (36.43)	482,475 (17.97)	899,402 (35.11)	233,971 (20.96)
	40s	1,856,180 (35.09)	1,130,178 (42.09)	727,029 (28.38)	444,828 (39.85)
	50s	1,155,569 (21.84)	1,003,132 (37.36)	421,101 (16.44)	380,754 (34.11)
**Participation in health checkups, n (%)**					
	No	1,065,901 (20.15)	1,939,509 (72.23)	502,002 (19.60)	753,052 (67.46)
	Yes	4,224,048 (79.85)	745,658 (27.77)	2,059,331 (80.40)	363,215 (32.54)
**Income quartile, n (%)**					
	Quartile 1 (high)	1,558,177 (29.46)	888,442 (33.09)	256,561 (10.02)	208,952 (18.72)
	Quartile 2 (high-middle)	1,605,305 (30.35)	574,625 (21.40)	532,219 (20.78)	201,514 (18.05)
	Quartile 3 (low-middle)	1,383,142 (26.15)	467,842 (17.42)	840,472 (32.81)	190,250 (17.04)
	Quartile 4 (low)	743,325 (14.05)	754,258 (28.09)	932,081 (36.39)	515,551 (46.19)
**Residential area, n (%)**					
	Metropolitan	2,763,458 (52.24)	1,363,397 (50.78)	1,395,343 (54.48)	589,891 (52.84)
	Other	2,526,491 (47.76)	1,321,770 (49.22)	1,165,990 (45.52)	526,376 (47.16)
**Disability, n (%)**					
	No	5,125,822 (96.9)	2,534,382 (94.38)	2,534,535 (98.95)	1,090,259 (97.67)
	Yes	164,127 (3.1)	150,785 (5.62)	26,798 (1.05)	26,008 (2.33)
**Charlson Comorbidity Index score, n (%)**					
	0	3,771,302 (71.29)	1,898,120 (70.69)	1,767,569 (69.01)	717,565 (64.28)
	1	1,045,956 (19.77)	491,244 (18.29)	572,112 (22.34)	254,572 (22.81)
	≥2	472,691 (8.94)	295,803 (11.02)	221,652 (8.65)	144,130 (12.91)

### Cumulative Incidence and Risk of All-Cause Mortality and Secondary Outcomes by Employment Status

**Figure 2.**
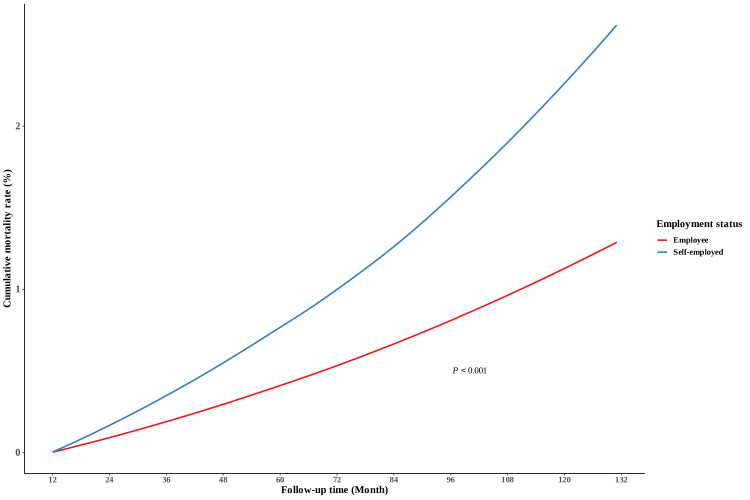
Age-standardized cumulative incidence of all-cause mortality by employment status among working individuals aged 20 to 59 y in the retrospective cohort, who were recruited from 2008 to 2010 (sourced from the National Health Insurance Service database in Korea; n=11,652,716).

During the median follow-up of 10.92 (IQR 10.92-10.92) years, all-cause mortality occurred in 1.93% (224,505/11,652,716) of participants—1.27% (99,542/7,851,282) of paid employees and 3.29% (124,963/3,801,434) of self-employed individuals (*P*<.001). The secondary outcome events were as follows: ischemic heart disease in 90,155 (1.15%) employees and 75,780 (1.99%) self-employed individuals; ischemic stroke in 80,646 (1.03%) employees and 83,481 (2.20%) self-employed individuals; and all types of malignancies (except thyroid cancer) in 259,724 (3.31%) employees and 179,706 (4.73%) self-employed individuals. The age-standardized cumulative 5- and 10-year incidence rates of all-cause mortality differed significantly by employment status (0.4% and 1.1%, respectively, for employees vs 0.8% and 2.3%, respectively, for self-employed individuals; *P*<.001; Figure 2). Regarding the secondary outcomes, there were significant differences (all *P* values were <.001) in 5- and 10-year age-cumulative incidence rates for all types of malignancies (thyroid cancer excluded from analysis; employees: 1% and 2.8%, respectively; self-employed individuals: 1% and 3%, respectively), ischemic heart disease (employees: 0.3% and 1%, respectively; self-employed individuals: 0.4% and 1.2%, respectively), ischemic stroke (employees: 0.3% and 0.9%, respectively; self-employed individuals: 0.5% and 1.3%, respectively), and hospitalization due to mental illness (employees: 0.5% and 1.1%, respectively; self-employed individuals: 1% and 2.2%, respectively; Multimedia Appendix 2).

The risk of all-cause mortality among the self-employed individuals, when compared to that among employees, was significantly higher, particularly among female individuals, according to the final model (male: adjusted HR 1.44, 95% CI 1.42‐1.45; female: adjusted HR 1.89, 95% CI 1.84‐1.94; *P*<.001; Table 2). With regard to the secondary outcomes, the risk of ischemic heart disease (adjusted HR 1.18, 95% CI 1.17-1.20; *P*<.001) and ischemic stroke (adjusted HR 1.34, 95% CI 1.33-1.36; *P*<.001) was significantly higher among the self-employed individuals than among employees, while the risk of all types of malignancies did not differ significantly by employment status (adjusted HR 1.01, 95% CI 0.99‐1.01; *P*=.09; Multimedia Appendix 3). Similar results were observed when the risk of secondary outcomes was compared between self-employed individuals and employees by sex subgroup.

**Table 2.** Adjusted HRs^a^ and 95% CIs for all-cause mortality, which was compared between self-employed individuals and employees among working individuals aged 20 to 59 y in the retrospective cohort, who were recruited from 2008 to 2010 (sourced from the National Health Insurance Service database in Korea; n=11,652,716).

Self-employed vs employee		Crude model, adjusted HR (95% CI)	Model 1^b^, adjusted HR (95% CI)	Model 2^c^, adjusted HR (95% CI)	Final model^d^, adjusted HR (95% CI)
Total^e^		2.62 (2.60‐2.64)	1.94 (1.92‐1.96)	1.80 (1.79‐1.82)	1.50 (1.48‐1.51)
Male		2.47 (2.45‐2.49)	1.88 (1.86‐1.90)	1.71 (1.69‐1.72)	1.44 (1.42‐1.45)
Female		3.09 (3.03‐3.16)	2.35 (2.29‐2.40)	2.37 (2.32‐2.42)	1.89 (1.84‐1.94)

a HR: hazard ratio.

b Model 1: adjusted for age.

c Model 2: adjusted for age, residential area, income, and disability.

d Model 3: adjusted for age, residential area, income, disability, health checkup participation, and Charlson Comorbidity Index score.

e Sex was adjusted in all models for the entire cohort.

### Subgroup and Sensitivity Analyses

The subgroup analysis results for male and female individuals are presented in Tables3  4, respectively. The analyses showed that all-cause mortality risk was significantly higher among self-employed individuals when compared to that among employees (*P*<.001). The reverse trends in adjusted HRs (and their 95% CIs) for the association between mortality and employment status in age, health checkup participation, and income level subgroups were consistent for both male individuals and female individuals. Additionally, the difference in all-cause mortality between self-employed individuals and employees was lower in the health checkup participation group. Further, self-employed participants in their 20s had a significantly higher risk of mortality when compared to employees in their 20s (*P*<.001). In the sensitivity analysis with different time lags and the exclusion of income-level outliers, similar results were reproduced (Multimedia Appendix 4). In the effect modification analysis, health checkup participation acted as an effect modifier for the association between employment status and all-cause mortality in both sexes (male: RERI 0.76, 95% CI 0.74‐0.79; female: RERI 1.13, 95% CI 1.05‐1.21; Multimedia Appendix 5).

**Table 3.** Subgroup analysis of the association between employment status and mortality among working male individuals aged 20 to 59 y in the retrospective cohort, who were recruited from 2008 to 2010 (sourced from the National Health Insurance Service database in Korea; n=7,975,116).

Subgroups		Self-employed			Employee			Adjusted HR^a^ (95% CI)
		Deaths, n	Person-years	Rate^b^	Deaths, n	Person-years	Rate^b^	
**Age group (y)**								
	20s	1697	3,826,451	44.35	910	753,563	120.76	2.61 (2.32‐2.94)
	30s	13,402	20,976,162	63.89	8790	5,230,214	168.06	1.88 (1.81‐1.95)
	40s	28,519	20,138,560	141.61	37,958	12,181,101	311.61	1.48 (1.45‐1.50)
	50s	41,107	12,437,679	330.50	57,457	10,716,148	536.17	1.32 (1.30‐1.34)
**Participation in health checkups**								
	No	64,124	45,833,069	139.91	21,248	8,056,908	263.72	1.79 (1.76‐1.82)
	Yes	20,601	11,545,784	178.43	83,867	20,824,118	402.74	1.12 (1.11‐1.14)
**Income quartile**								
	Quartile 1 (high)	21,204	16,918,536	125.33	19,573	9,630,239	203.25	1.16 (1.14‐1.19)
	Quartile 2 (high-middle)	21,635	17,429,729	124.13	17,216	6,206,800	277.37	1.18 (1.15‐1.21)
	Quartile 3 (low-middle)	22,905	14,999,216	152.71	19,288	5,026,951	383.69	1.40 (1.37‐1.43)
	Quartile 4 (low)	18,981	8,031,372	236.34	49,038	8,017,036	611.67	1.90 (1.86‐1.94)
**Residential area**								
	Metropolitan	40,507	29,991,089	135.06	50,357	14,676,008	343.12	1.50 (1.47‐1.52)
	Other	44,218	27,387,764	161.45	54,758	14,205,018	385.48	1.38 (1.36‐1.40)
**Disability**								
	No	79,097	55,612,295	142.23	95,468	27,275,642	350.01	1.45 (1.44‐1.47)
	Yes	5628	1,766,558	318.59	9647	1,605,384	600.92	1.27 (1.22‐1.32)
**Charlson Comorbidity Index score**								
	0	53,320	40,938,624	130.24	69,138	20,439,341	338.26	1.45 (1.43‐1.47)
	1	17,349	11,342,423	152.96	17,631	5,290,113	333.28	1.39 (1.35‐1.42)
	≥2	14,056	5,097,806	275.73	18,346	3,151,572	582.12	1.48 (1.44‐1.51)

a HR: hazard ratio.

b Per 100,000 person-years.

**Table 4.** Subgroup analysis of the association between employment status and mortality among working female individuals aged 20 to 59 y in the retrospective cohort, who were recruited from 2008 to 2010 (sourced from the National Health Insurance Service database in Korea; n=3,677,600).

Subgroups		Self-employed			Employee			Adjusted HR^a^ (95% CI)
		Deaths, n	Person-years	Rate^b^	Deaths, n	Person-years	Rate^b^	
**Age group (y)**								
	20s	1203	5,603,840	21.47	751	615,929	121.93	4.50 (3.96‐5.12)
	30s	3267	9,804,439	33.32	3133	2,540,504	123.32	2.78 (2.60‐2.96)
	40s	4950	7,915,890	62.53	6868	4,827,348	142.27	1.79 (1.71‐1.86)
	50s	5397	4,574,619	117.98	9096	4,119,034	220.83	1.58 (1.52‐1.64)
**Participation in health checkups**								
	No	11,700	22,431,883	52.16	4904	3,945,256	124.30	2.44 (2.35‐2.54)
	Yes	3117	5,466,904	57.02	14,944	8,157,559	183.19	1.49 (1.44‐1.54)
**Income quartile**								
	Quartile 1 (high)	1580	2,794,234	56.55	2271	2,272,205	99.95	1.23 (1.14‐1.32)
	Quartile 2 (high-middle)	2182	5,800,670	37.62	2658	2,189,392	121.40	1.57 (1.46‐1.68)
	Quartile 3 (low-middle)	4086	9,157,886	44.62	3013	2,064,454	145.95	1.71 (1.61‐1.81)
	Quartile 4 (low)	6969	10,145,997	68.69	11,906	5,576,764	213.49	2.20 (2.12‐2.27)
**Residential area**								
	Metropolitan	7701	15,200,200	50.66	9960	6,397,602	155.68	1.89 (1.83‐1.96)
	Other	7116	12,698,587	56.04	9888	5,705,212	173.32	1.89 (1.82‐1.96)
**Disability**								
	No	14,404	27,608,023	52.17	18,731	11,823,712	158.42	1.89 (1.84‐1.94)
	Yes	413	290,764	142.04	1117	279,103	400.21	1.88 (1.66‐2.13)
**Charlson Comorbidity Index score**								
	0	9221	19,257,284	47.88	11,698	7,784,726	150.27	1.90 (1.83‐1.96)
	1	3486	6,230,579	55.95	4285	2,761,141	155.19	1.86 (1.76‐1.95)
	≥2	2110	2,410,924	87.52	3865	1,556,948	248.24	1.94 (1.83‐2.06)

a HR: hazard ratio.

b Per 100,000 person-years.

## Discussion

In this study, self-employed individuals had a significantly higher risk of mortality than employees (*P*<.001), after adjusting for age, sex, residential area, income level, disability, health checkup participation, and chronic disease status. Subgroup analyses confirmed a prominent association among female individuals, young individuals, those with low incomes, and those who did not participate in health checkups. Regarding secondary outcomes, the risk of ischemic heart disease and ischemic stroke was significantly higher among self-employed individuals than among the employees (*P*<.001); however, no significant difference was observed in the risk of all types of malignancies (thyroid cancer was excluded from analyses; *P*=.09).

The risk of all-cause mortality among the self-employed individuals was higher when compared to that among employees, which may have several explanations. Cho et al [21] found that the suicidal mortality risk in their self-employed group was significantly higher than that in their employee group, especially among female individuals. This may be due to the precarious nature of being self-employed, based on studies suggesting that an unstable employment environment is associated with an increased suicide risk [22,23]. Another study revealed that self-employed female individuals reported high time constraints and work-life balance challenges [24]. A prominently higher risk of suicidal mortality among self-employed female individuals could plausibly explain the elevated risk of all-cause mortality among the self-employed female participants in this study. Moreover, in this study, the proportion of self-employed female smokers was significantly higher than that of employed female smokers (*P*<.001), reflecting the difference in mortality risk between these two groups. Owing to a lack of information regarding the lifestyle habits of those who did not participate in health checkups, this finding cannot be generalized to the entire population. However, this study highlights the importance of smoking cessation among self-employed female individuals and necessitates future studies involving detailed participant information.

This study also found that self-employed individuals had significantly higher risks of ischemic stroke and heart disease than employees (*P*<.001). This may be explained by ambiguous work schedules, income instability, and related work stress among self-employed individuals [25,26]. A similar association was reported in a study by Krittanawong et al [27], who found that self-employment was significantly associated with coronary artery disease, stroke, heart failure, and hospitalization due to mental health. Moreover, nonparticipation in periodic health checkups may result in a higher risk of cardiovascular disease [28], based on the substantial difference in health checkup participation between self-employed individuals and employees. Regarding mental illness, Cho et al [21] found that the risk of suicide was significantly higher among self-employed young people (notably among female individuals) than among employees, which is consistent with our finding. Job instability may explain the strong association among self-employed female individuals [23]; they often have the additional role of being a housewife or caring for children, making it difficult for them to endure the pressure of job instability, as suggested by social role theory [29].

One of the main differences between the self-employed and employee populations may be in their access to health care. For instance, the accessibility of health checkups differed between the two groups. Korea has a system that requires workers to undergo a medical checkup every 2 years, but other countries may not have such a system. Owing to the lack of responsible employers, the self-employed group had a significantly lower rate of health checkup participation than the employee group (*P*<.001), based on the results of this study as well as a study conducted by Kim et al [30] that used a nationwide survey. Additionally, self-employed individuals may lose their earnings when participating in health checkups, whereas employees are usually granted paid leave to participate in health checkups. Therefore, policies that compensate self-employed individuals for the income lost due to health checkup participation are necessary. This is also supported by the subgroup analysis results of this study, which indicated that the health disparity gap among the self-employed individuals who participated in health checkups was significantly lower when compared to that among the self-employed individuals who did not participate in health checkups (*P*<.001). The effect modification of health checkup participation also supports the need for policies that promote health checkups among self-employed individuals.

Mortality risk was also significantly higher in the self-employed group than in the employee group for all income quantiles (*P*<.001). This may be explained by self-employed individuals’ income insecurity, unpredictable work environments, and vulnerability to economic change [31]. Income insecurity among self-employed individuals may result in stressful conditions and related health issues [32]. In particular, the mortality risk difference between self-employed individuals and employees was larger in the lowest income quantile than in other income quantiles. This may be explained by the larger income stability gap between the low-income and low-middle–income groups.

This study identified a higher mortality risk difference in younger age groups, with a linear trend. According to the healthy worker effect, the risk of all-cause mortality declines with age because individuals who are unhealthy may retire for medical reasons, and healthier individuals may flourish in the company [33] (the healthy worker survivor effect) [34]. Similarly, older self-employed individuals may be healthier because they have already endured stressful situations, including income instability and job precarity. Further studies related to the effects of self-employed individuals’ age and health should be conducted to clarify the inverse trend of association.

This study’s main strength is that it compared the mortality risk between self-employed individuals and paid employees in terms of socioeconomic aspects, specifically occupation. Moreover, it estimated the mortality risk in analyses stratified by sex and other socioeconomic and medical traits, and it explored subgroups with a higher risk of all-cause mortality. The importance of health checkups was highlighted, and the association between health checkup participation and employment status was demonstrated. Moreover, this study enrolled approximately 11.6 million participants via a nationwide database and was representative of the economically active population, as the NHIS covered 97.2% of the Republic of Korea’s population [35].

There are, however, a few limitations. First, the causal relationship between mortality risk and employment status could not be confirmed in this observational study. Second, an analysis of suicide or accidental death could not be performed due to a lack of information on the cause of death, which was missing in our data. Instead, we performed an analysis of the association between hospitalization due to mental illness and employment status; nonetheless, further studies are needed to investigate the differences in suicide rates and accidental death rates between self-employed individuals and employees. Therefore, well-designed prospective studies should be performed. Third, some participants’ employment statuses may have been inaccurate, as these were indirectly inferred from their insurance type; this may be particularly true for the extremely high–income and extremely low–income populations. This study attempted to overcome this limitation by excluding participants with income outliers. Fourth, although this study included participants who maintained the same insurance type for 3 years, the dynamic nature of job status should be considered in future studies. Fifth, unmeasured confounders may exist in the NHIS, including lifestyle habits and subjective health statuses. An additional sensitivity analysis was performed on those who participated in health checkups and had information about lifestyle habits; however, selection bias may have occurred due to self-employed participants’ lack of health checkup participation, necessitating further research. Finally, given the varying differences in socioeconomic issues and characteristics between different countries, the results on self-employed individuals’ health should be interpreted cautiously when generalizing the findings to other countries’ populations.

In conclusion, this study found significantly increased risks of all-cause mortality, cardio-cerebrovascular diseases, and mental illnesses in self-employed individuals when compared to those among employees. This association was prominent among younger individuals, those with lower incomes, and those who did not participate in health checkups. Additionally, health checkup nonparticipation acted as an effect modifier for the association between employment status and the risk of all-cause mortality. This effect modification may stem from the differences in risks associated with cardiovascular and mental illnesses. Considering the health gap between the self-employed and employee populations in Korea, future studies and policies should aim to clarify substantial hazards to self-employed individuals’ health.

## Supplementary material

10.2196/48047 Multimedia Appendix 1 *International Statistical Classification of Diseases, Tenth Revision* (*ICD-10*) codes used for defining the Charlson Comorbidity Index (CCI) in this study with working individuals aged 20 to 59 years in the retrospective cohort, who were recruited from 2008 to 2010 (sourced from the National Health Insurance Service database in Korea; n=11,652,716).

10.2196/48047 Multimedia Appendix 2 Age-standardized cumulative incidence rates (by employment status) of (A) all types of malignancies (except thyroid cancer), (B) ischemic heart disease, (C) ischemic stroke, and (D) hospitalization due to a mental illness among working individuals aged 20 to 59 years in the retrospective cohort, who were recruited from 2008 to 2010 (sourced from the National Health Insurance Service database in Korea; n=11,652,716).

10.2196/48047 Multimedia Appendix 3 Adjusted hazard ratios (HRs) and 95% CIs for the secondary outcomes, which were compared between self-employed individuals and employees among working individuals aged 20 to 59 years in the retrospective cohort, who were recruited from 2008 to 2010 (sourced from the National Health Insurance Service database in Korea; n=11,652,716).

10.2196/48047 Multimedia Appendix 4 Sensitivity analyses on the risk of all-cause mortality, by employment status, among working individuals aged 20 to 59 years in the retrospective cohort, who were recruited from 2008 to 2010 (sourced from the National Health Insurance Service database in Korea; n=11,652,716).

10.2196/48047 Multimedia Appendix 5 Effect modification of health checkup participation on the association between employment status and all-cause mortality among working individuals aged 20 to 59 years in the retrospective cohort, who were recruited from 2008 to 2010 (sourced from the National Health Insurance Service database in Korea; n=11,652,716).

## Abbreviations

CCI: Charlson Comorbidity Index
HR: hazard ratio
*ICD-10*: *International Statistical Classification of Diseases, Tenth Revision*
NHIS: National Health Insurance Service
OECD: Organisation for Economic Co-operation and Development
RERI: relative excess risk due to interaction
