# Supplementary material for: Inequality in Mortality and Cardiovascular Risk Among Young, Low-Income, Self-Employed Workers: Nationwide Retrospective Cohort Study
Source: JMIR Public Health Surveill. 2024 Sep 20;10:e48047. doi: 10.2196/48047 (PMC11429069; doi:10.2196/48047)
Supplement: Multimedia Appendix 1 [file publichealth-v10-e48047-s001.docx]

| **Multimedia Appendix 1. ICD-10 code used for defining Charlson Comorbidity Index (CCI) in this study with working individuals aged 20–59 in the retrospective cohort recruited from 2008–2010, sourced from National Health Insurance Service Database in Korea (n=11,652,716)** | | |  |
| --- | --- | --- | --- |
| **Category** | **Diagnosis** | **ICD-10 code** | **Score** |
| Disease for Exclusion Based on CCI | Diabetes mellitus | E10–E14 | 1 |
|  | Myocardial infarct | I21, I22, I25 | 1 |
|  | Congestive heart failure | I50 | 1 |
|  | Peripheral vascular disease | I70–I79 | 1 |
|  | Cerebrovascular disease | I60–I69 | 1 |
|  | Dementia | F03, G30 | 1 |
|  | Chronic pulmonary disease | J41, J42, J43, J44, J45, J47, J64 | 1 |
|  | Rheumatic or connective tissue disease | M30–M36, M06 | 1 |
|  | Gastric or peptic ulcer | K25, K26 | 1 |
|  | Mild liver disease | B18, B19, K70–K77 | 1 |
|  | Hemiplegia or paraplegia | G80, G81, G82 | 2 |
|  | Moderate or severe renal disease | N17–N19 | 2 |
|  | Any malignancy, including lymphoma and leukemia, except basal cell cancer of skin^§^ | C00–C41, C43, C45–C72, C74, C75, C81–C96 | 2 |
|  | Metastatic solid tumor | C76–C80 | 6 |
|  | Acquired immune deficiency syndrome | B20-B24 | 6 |
| ^§^Thyroid cancer was excluded. | |  |  |
