# Supplementary figures and images for: Inequality in Mortality and Cardiovascular Risk Among Young, Low-Income, Self-Employed Workers: Nationwide Retrospective Cohort Study
Source: JMIR Public Health Surveill. 2024 Sep 20;10:e48047. doi: 10.2196/48047 (PMC11429069; doi:10.2196/48047)

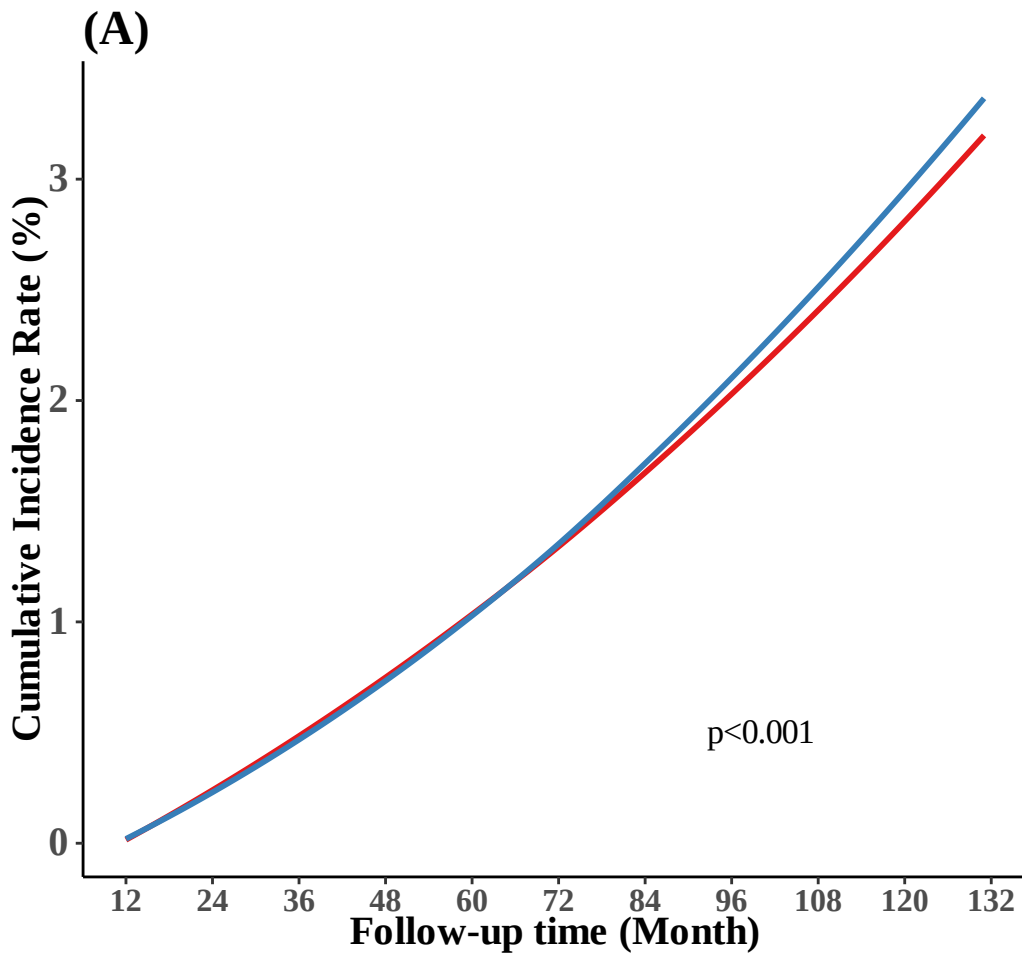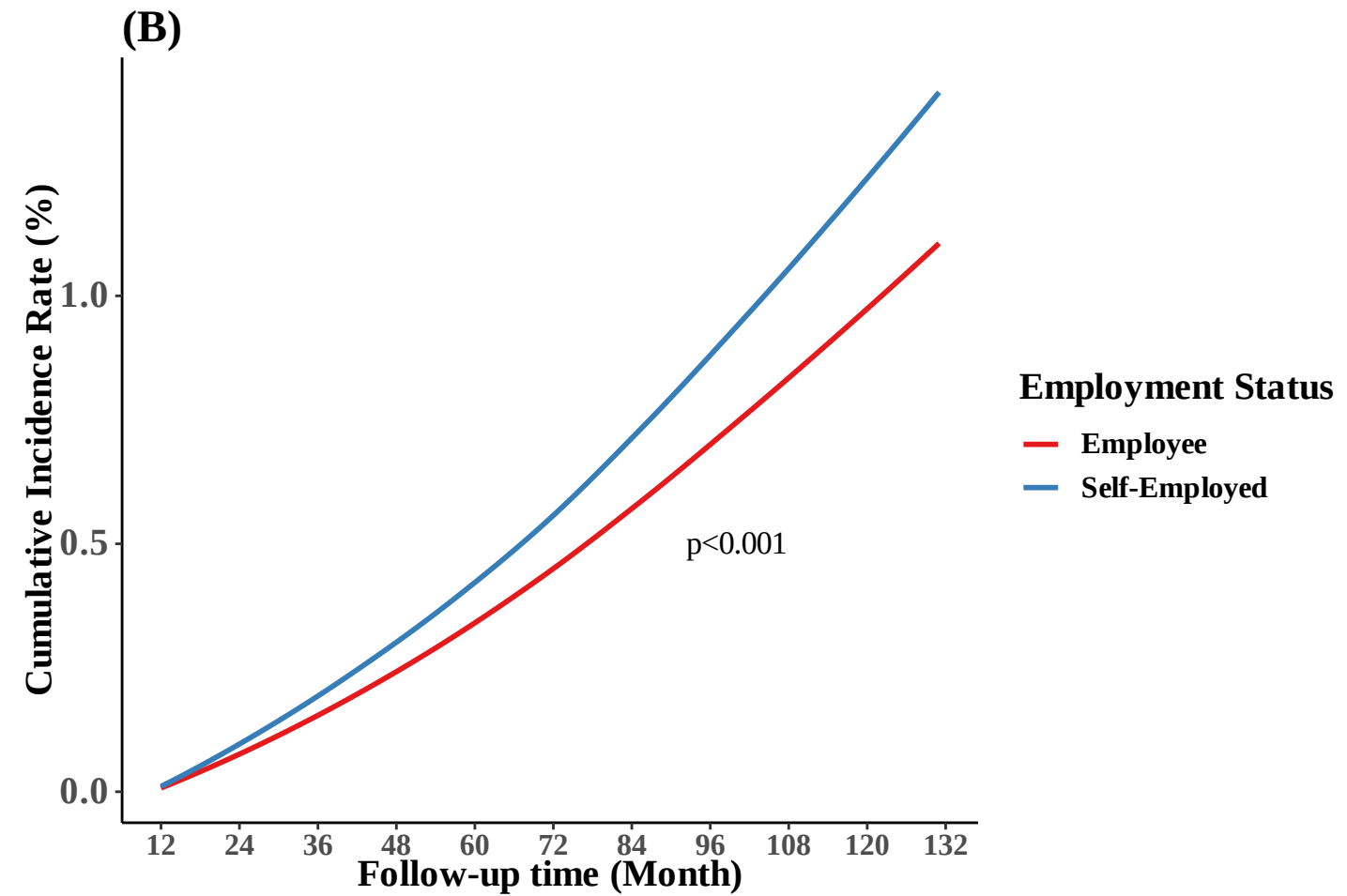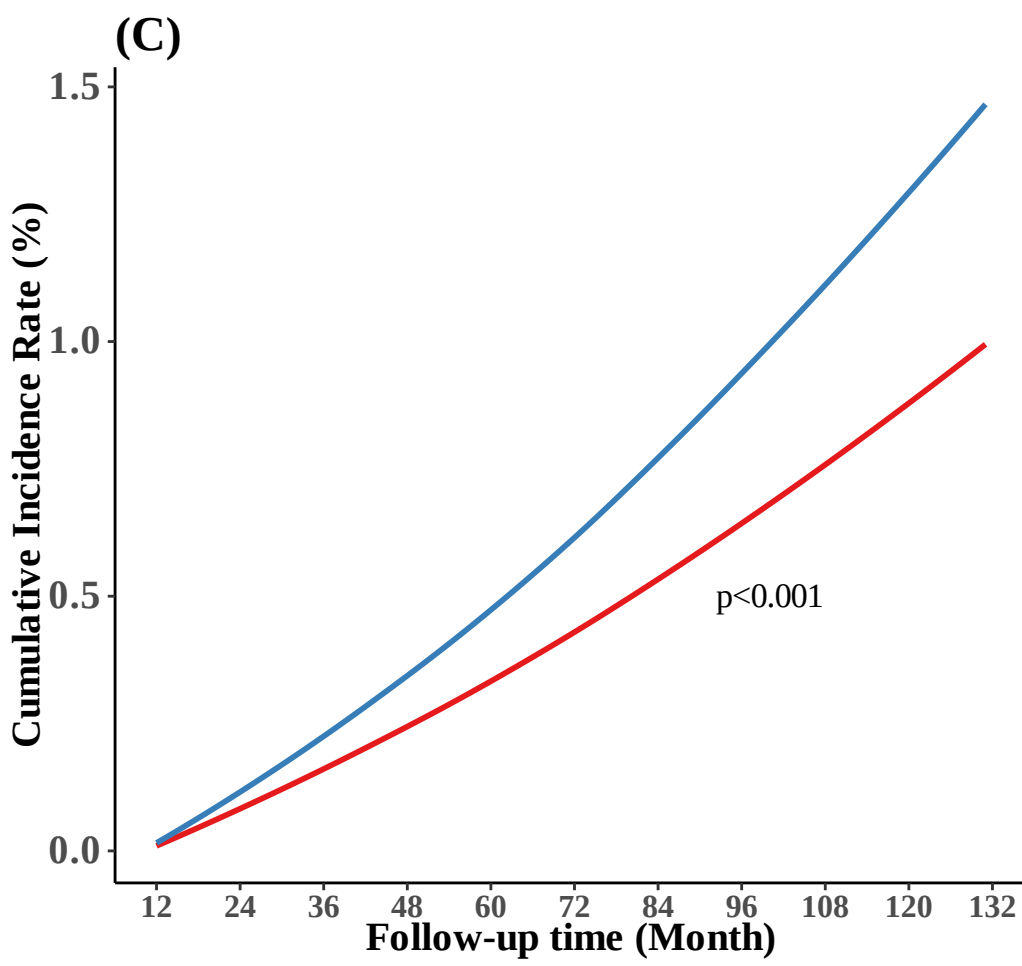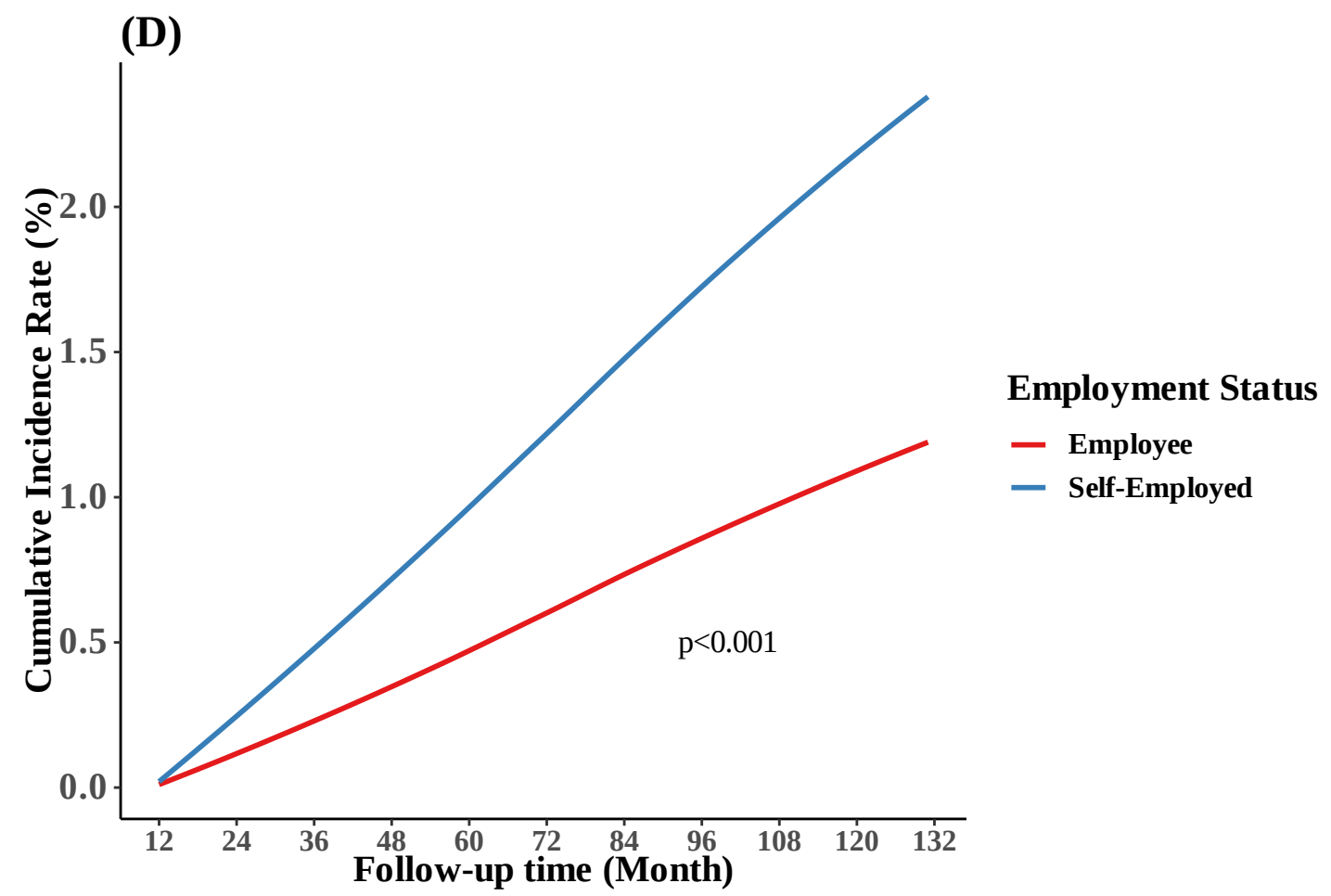

Supplement: Multimedia Appendix 2 [file publichealth-v10-e48047-s002.pdf]
