# Supplementary material for: Inequality in Mortality and Cardiovascular Risk Among Young, Low-Income, Self-Employed Workers: Nationwide Retrospective Cohort Study
Source: JMIR Public Health Surveill. 2024 Sep 20;10:e48047. doi: 10.2196/48047 (PMC11429069; doi:10.2196/48047)
Supplement: Multimedia Appendix 3 [file publichealth-v10-e48047-s003.docx]

| **Multimedia Appendix 3. Adjusted HR (95% CI) for the secondary outcomes comparing self-employed individuals to employees among working individuals aged 20–59 in the retrospective cohort recruited from 2008–2010, sourced from National Health Insurance Service Database in Korea (n=11,652,716).** | | | | | | | | |
| --- | --- | --- | --- | --- | --- | --- | --- | --- |
| **Type of insurance** | | **Ischemic heart disease** | | **Ischemic stroke** | **All types of malignancy^§^** | | **Hospitalization due to**  **A mental illness** | |
| **self-employed (vs. employee)** | **Total^¶^** | 1.18 (1.17-1.20) | | 1.34 (1.33-1.36) | 1.00 (0.99-1.01) | | 1.71 (1.69-1.73) | |
|  | **Men** | 1.15 (1.14-1.17) | | 1.31 (1.29-1.32) | 1.02 (1.01-1.02) | | 1.59 (1.57-1.61) | |
|  | **Women** | 1.37 (1.33-1.41) | | 1.51 (1.47-1.55) | 1.01 (0.99-1.02) | | 1.93 (1.89-1.96) | |
| Abbreviation: HR, hazard ratio; CI, confidence interval | | |  |  | |  | |  |
| ¶ Sex was adjusted in all models from the entire cohort | | |  |  | |  | |  |
| § Thyroid cancer was not included | |  |  |  | |  | |  |
