# Supplementary material for: Inequality in Mortality and Cardiovascular Risk Among Young, Low-Income, Self-Employed Workers: Nationwide Retrospective Cohort Study
Source: JMIR Public Health Surveill. 2024 Sep 20;10:e48047. doi: 10.2196/48047 (PMC11429069; doi:10.2196/48047)
Supplement: Multimedia Appendix 4 [file publichealth-v10-e48047-s004.docx]

| **Multimedia Appendix 4. Sensitivity analyses on the risk of all-cause mortality by employment status among working individuals aged 20–59 in the retrospective cohort recruited from 2008–2010, sourced from National Health Insurance Service Database in Korea (n=11,652,716).** | | | | |
| --- | --- | --- | --- | --- |
|  |  | **Total** | **Men** | **Women** |
| **Time lag for mortality (year(s))** | **1** | 1.52 (1.50-1.53) | 1.46 (1.44-1.47) | 1.88 (1.83-1.93) |
|  | **2** | 1.54 (1.52-1.55) | 1.48 (1.44-1.50) | 1.88 (1.83-1.93) |
|  | **3** | 1.55 (1.54-1.57) | 1.50 (1.48-1.52) | 1.89 (1.84-1.95) |
|  | **4** | 1.58 (1.56-1.59) | 1.52 (1.50-1.54) | 1.91 (1.85-1.96) |
| **excluding income outlier** | | 1.50 (1.49-1.52) | 1.44 (1.43-1.46) | 1.90 (1.85-1.95) |
| Values were expressed by adjusted hazard ratio (95% confidence interval) | | | |  |
| All models were adjusted by age, residential area, income, disability, health check-up participation, and charlson comorbidity index | | | | |
