# Supplementary material for: Inequality in Mortality and Cardiovascular Risk Among Young, Low-Income, Self-Employed Workers: Nationwide Retrospective Cohort Study
Source: JMIR Public Health Surveill. 2024 Sep 20;10:e48047. doi: 10.2196/48047 (PMC11429069; doi:10.2196/48047)
Supplement: Multimedia Appendix 5 [file publichealth-v10-e48047-s005.docx]

| **Multimedia Appendix 5. Effect modification of health check-up participation on the association between employment status and all-cause mortality among working individuals aged 20–59 in the retrospective cohort recruited from 2008–2010, sourced from National Health Insurance Service Database in Korea (n=11,652,716).** | | | | | | |  |
| --- | --- | --- | --- | --- | --- | --- | --- |
| **Employment status** |  | **Employee** | | **Self-employed** | | **RERI** | |
|  |  | **N with/without Health Check-Ups** | **HR(95% CI)** | **N with/without Health Check-Ups** | **HR(95% CI)** |  |  |
| **Health Check-Up**  **Participation (Male)** | **Yes** | 21,248/8,056,908 | 1.00 (Ref) | 64,124/45,833,069 | 1.14 (1.12-1.16) | 0.76 (0.74-0.79) | |
|  | **No** | 83,867/20,824,118 | 1.16 (1.14-1.17) | 20,601/11,545,784 | 2.06 (2.04-2.08) |  |  |
| **Health Check-Up**  **Participation (Female)** | **Yes** | 4,904/3,945,256 | 1.00 (Ref) | 11,700/22,431,883 | 1.56 (1.51-1.62) | 1.13 (1.05-1.21) | |
|  | **No** | 14,944/8,157,559 | 1.21 (1.17-1.27) | 3,117/5,466,904 | 2.91 (2.84-2.98) |  |  |
| Abbreviation: HR, hazard ratio; CI, confidence interval; RERI, relative excess risk due to Interaction | | | | | |  | |
| All models were adjusted by age, residential area, income, disability, health check-up participation, charlson comorbidity index, obesity, smoking, alcohol consumption, and physical activity | | | | | | | |
